# Seed-Guided *De Novo* Design Expands the Structural Diversity of Antitoxin Protein Binders

**DOI:** 10.64898/2026.08.02.742339

**Published:** 2026-08-04

**Authors:** Dustin Britton, Dia A Ghose, Jackson C Halpin, Foster Birnbaum, Kruthi Gundu, Shaunak Raval, Malvina Papanastasiou, Steven A Carr, Amy E. Keating

## Abstract

*De novo* design of protein binders targeting extended, multi-site interaction surfaces remains difficult for current generative methods, which often produce limited structural diversity and predominantly helical topologies. Here, we advance diffusion-based binder design by guiding protein backbone generation through ″seeds,″ which are PDB-derived fragments selected for geometric complementarity to the target surface. To test this approach, we computationally generated seed-guided binders of the bacterial toxin RelE. RelB, the native antitoxin of RelE, engages two distinct interfaces with high surface complementarity, making it an appropriate test case. Seed-guided RFdiffusion produced backbones with substantially higher structural diversity and more target contacts than RFdiffusion alone. Experimental screening of 1,402 designs in a high-throughput bacterial survival assay identified multiple functional binders, including variants with nanomolar to low-micromolar affinity and one design with RelE neutralization comparable to RelB _pep_ . Computational structure prediction and mutational analyses support that the designed interfaces rely on seed-derived contacts and adopt binding modes distinct from RelB. Molecular dynamics simulations and hydrogen-deuterium exchange experiments further suggest that one high-affinity design undergoes a conformational change upon binding. Notably, successful designs exhibited reduced cross-reactivity to RelE orthologs compared with RelB _pep_ , suggesting that the extensive interfaces generated through seed-guided design can enable enhanced selectivity. These results establish motif scaffolding of surface-complementing seeds as an effective strategy for overcoming current limitations of *de novo* generative models, enabling the design of proteins that can engage challenging interface sites.

## Full Text Availability

The license terms selected by the author(s) for this preprint version do not permit archiving in PMC. The full text is available from the preprint server.

